# Trends in caries experience in the permanent dentition in Germany 1997–2014, and projection to 2030: Morbidity shifts in an aging society

**DOI:** 10.1038/s41598-019-41207-z

**Published:** 2019-04-02

**Authors:** Rainer A. Jordan, Joachim Krois, Ulrich Schiffner, Wolfgang Micheelis, Falk Schwendicke

**Affiliations:** 1 Institute of German Dentists (IDZ), Cologne, Germany; 2 Department of Operative and Preventive Dentistry, Charité – Universitätsmedizin Berlin, Berlin, Germany; 3 Department of Restorative and Preventive Dentistry, University Medical Center Hamburg, Hamburg, Germany

## Abstract

The aims of this study were to assess the trends in dental caries experience in the permanent dentition (i.e., the number of decayed, missing, or filled teeth, DMFT) in Germany from 1997–2014 and to project caries experience to 2030. Components of caries experience (decayed teeth, DT, missing teeth, MT, filled teeth, FT) from repeated waves (1997, 2005, 2014) of the nationally representative German Oral Health Studies were analyzed in 12-, 35–44-, and 65–74-year-olds. Weighted means were interpolated cross-sectionally by fitting piecewise-cubic spline-curves and were then subjected to longitudinal regression and combined with population estimates. In 1997, children (12-year-olds) had a mean caries experience (decayed, missing, filled teeth, DMFT) of 1.7 teeth; this experience decreased to 0.5 teeth in 2014. For 2030, an experience of 0.2 teeth is projected. In adults (35–44-year-olds), a decrease was recorded (1997: 16.1 teeth; 2014: 11.2 teeth). This decrease is expected to continue until 2030 (to 7.7 teeth). Similarly, in seniors (65–74-year-olds), a decrease was recorded (1997: 23.6 teeth; 2014: 17.7 teeth); this decrease is expected to continue until 2030 (to 14.9 teeth). While the number of missing teeth has decreased consistently across age groups, the number of filled and decayed teeth has increased in seniors and is expected to continue to increase. The cumulative caries experience has decreased from 1.1 billion DMFT in 2000 to 867 million in 2015 and is expected to decrease to 740 million in 2030. Caries experience in the permanent dentition has been decreasing substantially, mainly due to a decrease in missing teeth. Younger age groups also show fewer decayed and filled teeth, while in older groups, restorative needs have not decreased, as more teeth are retained. Concepts for addressing the emanating morbidity shifts are required.

## Introduction

Dental caries prevalence and experience (i.e., the number of decayed, missing, filled teeth, DMFT) has been declining in many high-income countries for decades^1,2^, while globally, there is evidence of an increase in caries prevalence and experience^3^ and an increase in polarization, i.e., an unequal distribution of this prevalence and experience^4^. Untreated carious lesions in permanent teeth remain the most prevalent condition^5^, affecting 2.4 billion people worldwide in 2016. With nearly 33,000 cases per 100,000 inhabitants, caries is also the most prevalent condition in Germany^6^. As caries is the main reason for tooth loss up to the fifth decade of life^7,8^, the overall and long-term burden emanating from this condition can be assumed to be even higher^5^. The costs for treating dental caries are substantial^9^, as restorative and prosthetic interventions often require repeating given the limited lifespan of dental restorations and prostheses^10,11^.

The latest caries epidemiological data from Germany indicate a sustainable decline in caries experience in younger age groups, which is in line with data from other countries^12^. In parallel, the population is significantly aging; for example, individuals 65 years old and older comprise 22% of today’s population; this percentage will increase to 28% by 2030^13^. With more teeth retained in this growing age group, the absolute number of teeth at risk for dental caries is increasing as well. Both the morbidity and demographic dynamics of caries will have a significant impact on future treatment needs.

Evaluating time trends and projecting future caries experience is relevant for clinicians and for resource allocation. The aims of this study were to assess dental caries trends in the permanent dentition in Germany and to project caries experience to 2030.

## Material and Methods

### Data source and participants

Data were obtained from a repeated cross-sectional oral epidemiological survey, originally started in 1989 in Germany, called the German Oral Health Study (Deutsche Mundgesundheitsstudie, DMS). DMS involves stratified multistage cross-sectional, nationwide probability samples of the civilian German population, with clinical and socioepidemiological examinations in different age cohorts (12-year-olds, 35–44-year-olds, and 65–74-year-olds; and in DMS V, 75–100-year-olds). The DMS data collection waves (abbreviated “waves”) were as follows:

- Wave 1 data collection (DMS I): 1989, for the former Federal Republic of Germany (data not included in this study).
- Wave 2 data collection (DMS II): 1992, for the former German Democratic Republic (data not included in this study).
- Wave 3 data collection (DMS III): 1997, in Germany.
- Wave 4 data collection (DMS IV): 2005, in Germany.
- Wave 5 data collection (DMS V): 2014, in Germany.

The sampling design, data collection protocols and data availability can be found elsewhere^14–17^.

The study participants were drawn from local resident registration offices in 90 randomly selected communities (sample points) using cluster random sampling stratified for regions and areas of urbanization. A disproportional sample point selection was performed, with 60 study sample points in the Western federal states of Germany and 30 study sample points in the Eastern states. For DMS III, 3,065 participants were included (response rate of 63.6%); for DMS IV and V, these numbers were 4,631 (63.1%) and 4,609 (50.1%), respectively. Empirical nonresponder analyses were conducted to compare the sociodental characteristics of responders with those of the target population according to gender, educational level, dental visiting patterns, and dental/prosthetic status. Nonresponse bias was found to be minimal (appendix Tables S1–2). The study was ethically approved by the Medical Association North-Rhine (No. 2013384), as were all data collection protocols. All participants completed written informed consent forms. All methods were performed in accordance with the relevant guidelines and regulations.

### Caries examinations

The dental examinations and the socioscientific survey were carried out at the local sample points. To ensure reproducibility, interviewers and dental investigators were trained and calibrated by experts, and multiple reliability checks were performed throughout the field phase.

Dental examinations were performed by four teams working in parallel. Each team consisted of one dentist, one interviewer, and one contact person. Dental caries experience in the permanent dentition (DT, MT, FT) was recorded on 28 teeth (i.e., third molars were excluded) on five surfaces per posterior tooth (premolars and molars) and four surfaces per anterior tooth (incisors and canines). Teeth needed to be erupted at least beyond the anatomic tooth equator (i.e., the maximum circumference of the tooth crown) to be assessed. Caries findings were examined visually (not through exploration with a dental probe) according to WHO recommendations for epidemiological field studies^18^. Only a blunt periodontal probe (PCP 11.5B, HuFriedy, Tuttlingen) was used to assess restoration defects. On proximal surfaces, the contact with the neighboring tooth frequently made it difficult to conclusively detect carious lesions. In a modification of the WHO standards, the dental investigators recorded DT if typical signs of a proximal lesion were evident. Dental restorations were recorded in all cases where a carious lesion was assumed to be the reason for the placement of this restoration. If at least one tooth surface was carious or filled, the whole tooth was classified as either carious or filled. If both a carious lesion and a dental restoration were present on the same tooth, it was recorded as carious if the lesion extended into dentin; otherwise, it was recorded as restored.

Further recorded clinical parameters were periodontal diseases, prosthodontic status, developmental and acquired dental hard tissue defects. A paper-based questionnaire was completed by the subjects before the clinical examination, comprising questions on oral hygiene habits/prosthesis hygiene, the utilization of dental services, childhood and course of life, and social demographics, including place of residence and place of birth.

### Cross-sectional imputation

In the three waves of the DMS, patient data were available for particular age cohorts (12-year-olds, 35–44-year-olds and 65–74-year-olds). The mean decayed teeth (DT), mean missing teeth (MT) and mean filled teeth (FT) were computed for each of these age groups in each DMS wave (DMS III, IV and V). Age groups not sampled by the DMS were interpolated cross-sectionally by fitting a piecewise cubic polynomial spline^19^ to the weighted means of the DT and FT, respectively. For the MT, a sigmoid was fitted to the weighted means of MT. Boundary conditions were set to zero for <6-year-olds for the DT and FT and for the MT for <13-year-olds. The DT, MT and FT were summed to obtain the DMFT; the maximal DMFT was 28.

### Longitudinal imputation

The nonlinear pattern of the DMFT per age group was modeled longitudinally for the period 1997 to 2030 by applying log-linearization and then fitting a linear regression model. Thereafter, exponentiation was applied to reproduce the nonlinear pattern.

### Population level estimates

The mean DMFT for each age group and year were combined with recorded (1997–2015) and predicted population estimates (2020–2030) to determine the overall absolute number of teeth with caries experience^20^. The so-called G1-L1-W2 scenario prediction model was used, assuming that fertility will remain nearly constant at the level of 1.4 children per woman (G1), that life expectancy will moderately increase to 84.8 years for men and to 88.8 for women (L1) and that until 2021, the migration balance decreases to 200,000 persons per year and remains constant thereafter (W2)^13^.

## Results

The mean caries experience and its components for the three waves of the DMS are displayed in Table 1.

**Table 1:** Mean caries experience (decayed, missing, filled teeth, DMFT, and its components) in DMS waves III (1997), IV (2005), and V (2014) and the 2030 projection for Germany.

Age group	Category	DMS III	DMS IV	DMS V	2030
12 years old	**DT**	0.4	0.2	0.1	0.0
	**MT**	0.0	0.0	0.1	0.1
	**FT**	1.3	0.5	0.3	0.1
	**DMFT**	1.7	0.7	0.5	0.2
35–44 years old	**DT**	0.5	0.5	0.5	0.6
	**MT**	3.9	2.4	2.1	0.5
	**FT**	11.7	11.7	8.6	6.6
	**DMFT**	16.1	14.5	11.2	7.7
65–74 years old	**DT**	0.3	0.3	0.5	0.9
	**MT**	17.6	14.1	11.1	6.9
	**FT**	5.8	7.7	6.1	7.1
	**DMFT**	23.6	22.1	17.7	14.9

The mean number of teeth with caries experience decreased steadily in children (12 years old) between 1997 (1.7 teeth) and 2014 (0.5 teeth). Projected on all individuals in this age group, a decrease from 1.5 million teeth in 1997 to 0.35 million teeth in 2014 was noted. A further decrease in the mean number of teeth with caries experience in 2030 to 0.2 teeth in this age group is expected, which means a decrease to 0.1 million teeth (Table 1).

In younger adults (35–44 years old), the mean number of teeth with caries experience decreased steadily between 1997 (16.1 teeth) and 2014 (11.2 teeth). Projected on all individuals in this age group, a decrease from 204.4 million teeth in 1997 to 111.8 million teeth in 2014 was noted. A further decrease in the mean number of teeth with caries experience in 2030 to 7.7 DMF teeth is expected, which means a decrease to 82.5 million teeth in all individuals of this age group (Table 1).

In younger seniors (65–74 years old), the mean number of teeth with caries experience has likewise decreased steadily between 1997 (23.6 teeth) and 2014 (17.7 teeth). Projected on all individuals in this age group, a decrease from 174.2 million teeth in 1997 to 149.2 million teeth in 2014 was noted. A further decrease in the mean number of teeth with caries experience in 2030 to 14.9 DMF teeth is expected, which means an increase to 171.6 million teeth in all individuals of this age group (given that this group grows in absolute terms) (Table 1).

A population-wide projection of caries experience according to 10-year age strata is presented in Table 2.

**Table 2:** Mean and absolute number of decayed, missing, and filled teeth and caries experience (DMFT) projected for Germany, 2000–2030.

Year	Age group	DT	Decayed teeth (in millions)	MT	Missing teeth (in millions)	FT	Filled teeth (in millions)	DMFT	DMFT teeth (in millions)
2000	6–9 years old	0.1	0.338	0.1	0.350	0.3	0.913	0.5	1.600
	10–19 years old	0.3	2.804	0.3	2.515	1.7	16.069	2.3	21.388
	20–29 years old	0.4	4.040	0.7	6.494	6.2	59.410	7.3	69.944
	30–39 years old	0.5	6.665	1.7	23.222	10.7	148.323	12.8	178.211
	40–49 years old	0.5	5.467	3.9	46.759	11.3	136.429	15.6	188.656
	50–59 years old	0.4	3.655	8.0	78.358	9.6	94.201	17.9	176.214
	60–69 years old	0.3	2.886	13.6	134.775	7.3	72.074	21.2	209.735
	70–79 years old	0.3	1.631	19.1	123.289	5.4	35.018	24.8	159.938
	80–89 years old	0.2	0.590	22.8	58.294	3.7	9.435	26.7	68.320
	≥90 years old	0.2	0.118	24.7	12.974	1.9	0.980	26.8	14.072
	cum (in millions)		28.194		487.030		572.852		1088.078
2005	6–9 years old	0.1	0.217	0.1	0.267	0.2	0.560	0.3	1.044
	10–19 years old	0.2	2.023	0.2	1.873	1.3	11.360	1.7	15.256
	20–29 years old	0.4	3.649	0.5	5.074	5.3	51.917	6.2	60.640
	30–39 years old	0.5	5.539	1.3	14.950	9.6	112.440	11.4	132.930
	40–49 years old	0.5	6.499	3.0	40.357	10.4	140.436	13.8	187.292
	50–59- years old	0.4	4.363	6.3	66.109	9.1	95.179	15.8	165.651
	60–69 years old	0.3	3.472	11.4	114.726	7.3	73.175	19.1	191.373
	70–79 years old	0.3	2.131	17.0	116.118	5.6	38.391	23.0	156.640
	80–89 years old	0.3	0.910	21.4	65.776	3.9	12.041	25.6	78.727
	≥90 years old	0.3	0.177	24.0	14.496	2.0	1.188	26.2	15.861
	cum (in millions)		28.980		439.746		536.687		1005.414
2015	6–9 years old	0.0	0.086	0.1	0.148	0.1	0.202	0.2	0.436
	10–19 years old	0.1	1.029	0.1	1.005	0.7	5.584	1.0	7.619
	20–29 years old	0.3	2.990	0.3	3.101	4.0	39.741	4.6	45.832
	30–39 years old	0.5	4.701	0.8	7.600	7.8	79.271	9.0	91.573
	40–49 years old	0.5	6.248	1.8	20.432	8.8	101.147	11.1	127.827
	50–59 years old	0.5	6.833	4.0	51.705	8.2	106.771	12.7	165.309
	60–69 years old	0.5	4.613	8.0	76.341	7.3	69.243	15.8	150.198
	70–79 years old	0.5	3.946	13.6	111.671	6.1	49.990	20.1	165.606
	80–89 years old	0.5	1.957	18.9	75.716	4.4	17.725	23.8	95.398
	≥90 years old	0.5	0.361	22.6	16.246	2.2	1.570	25.3	18.176
	cum (in millions)		32.764		363.965		471.244		867.974
2030	6–9 years old	0.0	0.025	0.0	0.073	0.0	0.052	0.1	0.150
	10–19 years old	0.1	0.431	0.1	0.444	0.3	2.244	0.4	3.118
	20–29 years old	0.2	1.725	0.1	1.140	2.6	20.565	2.9	23.431
	30–39 years old	0.5	4.428	0.3	3.304	5.8	56.302	6.6	64.034
	40–49 years old	0.7	7.173	0.8	8.947	6.8	75.158	8.3	91.278
	50–59 years old	0.7	7.406	2.0	19.764	7.1	70.123	9.8	97.294
	60–69 years old	0.8	10.033	4.7	58.902	7.2	90.515	12.8	159.450
	70–79 years old	0.9	8.452	9.6	89.676	6.8	63.135	17.3	161.263
	80–89 years old	1.0	4.95	15.7	75.053	5.3	25.382	22.0	105.385
	≥90 years old	1.1	1.613	20.7	29.648	2.6	3.674	24.4	34.934
	cum (in millions)		46.236		286.951		407.150		740.337

A decrease in the cumulative caries experience to 740,337,000 DMF teeth in 2030 is expected for the entire German population. Similarly, an ongoing decrease is expected in filled teeth (2030: 407,150,000 FT) and missing teeth (2030: 286,951,000 MT). In contrast, the number of decayed teeth is expected to increase noticeably (2030: 46,236,000 DT).

**Figure 1:**
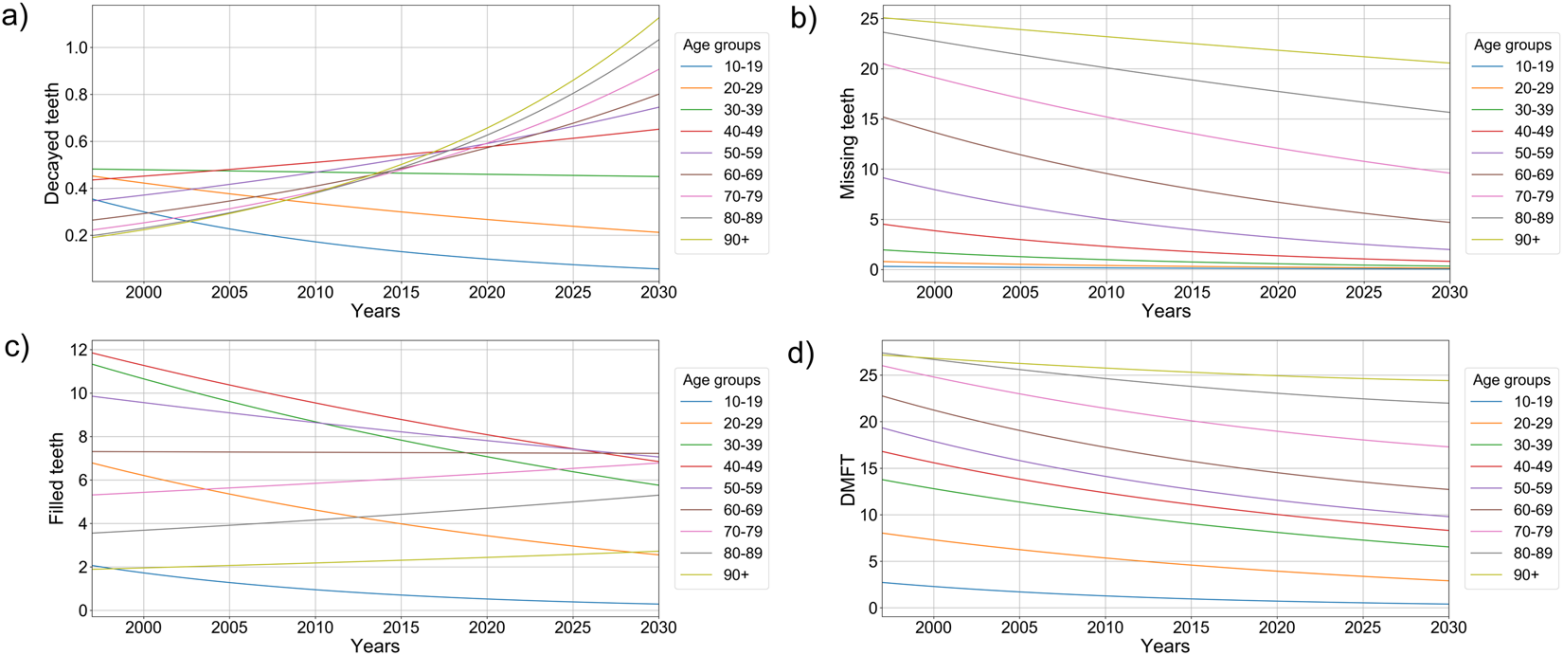
Number of teeth with caries experience (DMF) in different age groups and across time. (**a**) Mean number of decayed teeth between 1997 and 2030. (**b**) Mean number of missing teeth between 1997 and 2030. (**c**) Mean number of filled teeth between 1997 and 2030. (**d**) Mean number of DMF teeth between 1997 and 2030.

**Figure 2:**
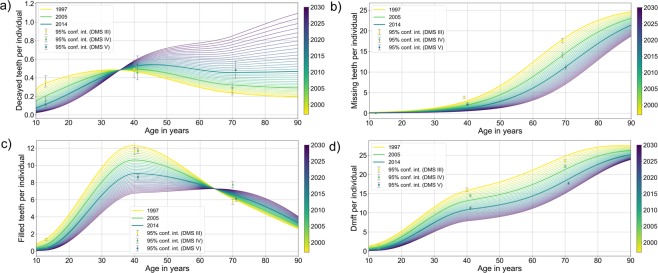
Number of teeth with caries experience (DMF) across ages in the years 1997–2030. (**a**) Development of decayed teeth according to age. (**b**) Development of missing teeth according to age. (**c**) Development of filled teeth according to age. (**d**) Cumulative DMF teeth according to age. The line colors indicate the year of data collection (note longitudinal interpolation); whiskers indicate the measured mean and 95% CI of the different indicators within the German Oral Health Studies (which allows for the assessment of the fit of the interpolation).

The underlying morbidity dynamic is illustrated in Fig. 1, which demonstrates the trends in caries experience and its components according to age groups between 1997 and 2030. Figure 2 displays these trends over all age groups. While overall caries experience and MT are expected to decrease further, DT and FT have decreased in younger age groups and increased in seniors and are expected to do so in the future. Especially for DT, an increase was observed (from the age of 36 years) and is expected in the future.

## Discussion

### Principal findings

Subsequent to the introduction of the wide availability of fluoridated toothpaste and oral prevention programs in kindergartens and schools as well as individual preventive measures in dental practices in Germany in the 1980s, the caries experience in younger age groups started to decline. In 12-year-old children, a decline in caries experience of approximately 70% from 1997 to today was recorded; this reduction is one of the most pronounced worldwide^21^. In absolute terms, the number of teeth with caries experience in this age group has decreased by a staggering 93%. In adults and seniors, tooth loss has similarly declined, as it is the major contributor to an overall decrease in caries experience. In adults, tooth loss was halved between 1997 and 2014, accompanied by a decline in filled teeth. In seniors, tooth loss has also decreased (by a total of 6.5 teeth), while the number of filled teeth has not declined.

For 2030, a continuing decrease in caries experience throughout all age groups was projected for Germany; mainly as a result of the decrease in missing teeth in older age groups and filled teeth in younger age groups. Notably, in older adults and seniors, this decrease will be accompanied by an increase in decayed and, to some degree, filled teeth, mainly as a result of more teeth being retained and now being at risk for caries. Given the described demographic shifts in Germany^22^, the cumulative number of decayed teeth but not the overall caries experience is expected to increase. Considering that these teeth need restorative treatment, this finding is noteworthy.

### Limitations

This study and the methodical approach have several limitations. First, applying log-linearization to model nonlinear patterns introduced some bias. Second, our extrapolation approach was based only on one covariate, the year of birth of an individual (in different age groups), assuming past changes per individual continuing to some degree in the future. Alternatively, a multivariate regression model for prediction was tested based on a set of socioeconomic variables and predictors of health behavior, but no improved explanation for the variance was found. The year of birth and the age of the individuals were the covariates with the greatest predictive power, allowing us to rely on such a parametric approach. Fourth, our data do not allow us to monitor the extent of the disease by tooth surface or to discriminate between shallow and deep carious lesions. Both types of lesions, however, may be of interest when considering that provided treatments may differ for differently extended or deep lesions (or restorations). Lastly, the number of noncavitated carious lesions in Germany was not estimated; these lesions are directly linked with noninvasive (preventive/nonoperative) treatment needs, which may also be of interest.

### Interpretation and implications

Our findings have implications on various levels. First, restorative treatment needs (i.e., those to manage decayed teeth and replace existing restorations) will be increasingly age-specific; the DT will decrease in populations aged <40 years, and the FT will decrease in populations aged <65 years, until 2030. In contrast, both the DT and FT are expected to increase in older age groups. Targeted approaches for preventing carious lesions and managing existing ones in these age groups may be needed in the future. Additionally, considering that the restoration spiral, i.e., the need for the repeated replacement of restorations^11^, will start later in life, restorative patterns of retreatment may generally change. Consequently, the diagnostic and operative skill of future dentists should be developed considering these changing patterns in morbidity and demography. Second, as dental caries is chronic in nature, the burden of disease is cumulative throughout the lifespan. The resulting concentration of morbidity in older age groups can be observed for dental caries^23^. Managing invasive dental treatments in the elderly, especially in case of long-term care and hospitalized patients, will become a major challenge for the dental profession, especially when considering possible comorbidities and sequelae of poor oral health^24,25^. In this respect, there is epidemiological evidence that, at present, the demand for oral health care in patients in need of care may not be fully met in Germany^15^. There is a need for the dental profession to develop comprehensive treatment concepts for this growing group.

## Conclusion

Based on the present study, dental caries experience in the permanent dentition has declined throughout all age groups in Germany since 1997 and is expected to decline further in the future. This decrease is mainly due to the decreasing number of missing teeth. In younger individuals, the number of decayed and filled teeth has also decreased, while in older individuals, there has been an increase in both, which is expected to be a stable future trend. With the concentration of caries experience in the growing group of elderly patients, restorative and prosthetic treatment needs will be concentrated in this age group. Our findings are a call to action for policymakers and researchers to address the consequences of these dynamics in individual and population health.

## Supplementary information

Appendix

## Data Availability

Data for the population projection 2030 in Germany are publicly available at the Federal Statistical Office (destatis.de). Data on the number of dentists in Germany can be obtained from the Statistical Yearbook of the German Dental Association (bzaek.de). The morbidity data are available as scientific reports from the Institut der Deutschen Zahnärzte (idz.institute).
