# Supplementary material for: Trends in caries experience in the permanent dentition in Germany 1997–2014, and projection to 2030: Morbidity shifts in an aging society
Source: Sci Rep. 2019 Apr 2;9:5534. doi: 10.1038/s41598-019-41207-z (PMC6445067; doi:10.1038/s41598-019-41207-z)
Supplement: Supplementary file 1 — Appendix [file 41598_2019_41207_MOESM1_ESM.docx]

Title:

**Trends in caries experience in the permanent dentition in Germany 1997-2030: Morbidity shifts in an aging society**

Contributing authors:

Prof. Dr. Rainer A. Jordan, MSc.^1*^

Joachim Krois^2^

Prof. Dr. Ulrich Schiffner^3^

Dr. Wolfgang Micheelis^1^

Priv.-Doz. Dr. Falk Schwendicke^2^

Appendix:

**Nonresponse analysis**

| Table S1: Nonresponse (NR) estimable model for adults (35-44 yr) | | | | | | | | | | | | |
| --- | --- | --- | --- | --- | --- | --- | --- | --- | --- | --- | --- | --- |
|  | DMS III (1997) | | | | DMS IV (2005) | | | | DMS V (2014) | | | |
|  | Subjects | NR subjects | Nonparticipants | total | Subjects | NR subjects | Nonparticipants | Total | Subjects | NR subjects | Nonparticipants | Total |
| No. of cases (n) | 655 | 243 | 281 | 1179 | 925 | 342 | 507 | 1774 | 966 | 348 | 673 | 1987 |
| Sampling rate (%) | 65 | 20 | 24 | 100 | 52 | 19 | 29 | 100 | 49 | 18 | 34 | 100 |
| Characteristics (%) | | | | | | | | | | | | |
| Male | 47,3 | 49,6 | ? | 48,3 | 50,6 | 54,7 | ? | 52,6 | 46,9 | 48,9 | ? | 48,4 |
| Low educational level | 28,1 | 30,0 | ? | 28,9 | 49,4 | 45,3 | ? | 47,7 | 53,1 | 51,1 | ? | 52,6 |
| Very good/good self-rated oral health status | 33,7 | 48,7 | ? | 40,3 | 40,6 | 53,2 | ? | 46,7 | 47,9 | 65,0 | ? | 57,3 |
| Regular dental check-ups | 68,9 | 62,1 | ? | 65,9 | 76,1 | 64,9 | ? | 70,7 | 76,7 | 67,6 | ? | 72,7 |
| Denture rate | 68,2 | 72,5 | ? | 70,1 | 68,1 | 63,2 | ? | 65,7 | 76,7 | 67,6 | ? | 72,7 |

| Table S2: Nonresponse (NR) estimable model for seniors (65-74 yr) | | | | | | | | | | | | |
| --- | --- | --- | --- | --- | --- | --- | --- | --- | --- | --- | --- | --- |
|  | DMS III (1997) | | | | DMS IV (2005) | | | | DMS V (2014) | | | |
|  | Subjects | NR subjects | Nonparticipants | total | Subjects | NR subjects | Nonparticipants | Total | Subjects | NR subjects | Nonparticipants | Total |
| No. of cases (n) | 1367 | 480 | 577 | 2424 | 1040 | 359 | 469 | 1868 | 1042 | 428 | 549 | 2019 |
| Sampling rate (%) | 56 | 20 | 24 | 100 | 56 | 19 | 25 | 100 | 52 | 21 | 27 | 100 |
| Characteristic (%) | | | | | | | | | | | | |
| Male | 44,7 | 33,4 | ? | 39,8 | 46,2 | 42,1 | ? | 44,4 | 47,0 | 40,9 | ? | 44,1 |
| Low educational level | 75,3 | 76,0 | ? | 44,0 | 65,8 | 62,6 | ? | 64,4 | 47,7 | 50,6 | ? | 49,1 |
| Very good/good self-rated oral health status | 47,1 | 39,96 | ? | 44,0 | 36,5 | 41,8 | ? | 38,8 | 45,6 | 51,2 | ? | 48,3 |
| Regular dental check-ups | 56,4 | 44,1 | ? | 51,0 | 72,2 | 70,9 | ? | 71,6 | 91,4 | 73,9 | ? | 83,0 |
| Denture rate |  |  |  |  |  |  |  |  |  |  |  |  |
